# Streff-Like Syndrome in an Adult: A Case Report

**DOI:** 10.7759/cureus.61289

**Published:** 2024-05-29

**Authors:** Khalilah Mastura Zahari, Siti Famira Rosland, Ch'ng Hannie, Othmaliza Othman

**Affiliations:** 1 Ophthalmology, Universiti Kebangsaan Malaysia Medical Centre, Kuala Lumpur, MYS; 2 Ophthalmology, Kuala Lumpur Hospital, Kuala Lumpur, MYS

**Keywords:** interdisciplinary management, psychogenic, non-organic visual loss, functional visual loss, streff syndrome

## Abstract

Streff syndrome is a non-malingering visual disturbance commonly affecting near and color vision, which is prevalent in the younger population. This case report presents Streff-like syndrome in a middle-aged woman. A 47-year-old woman with underlying major depressive disorder presented with bilateral reduced vision and a constricted visual field for one week. These symptoms were accompanied by additional neurological complaints of headache, weakness, and numbness triggered after a stressful event that affected her work performance. Examinations revealed reduced vision more toward near, diminished red saturation, color vision deficiencies, and bilateral tubular visual fields. Notably, the relative afferent pupillary defect was negative, with both anterior and posterior segments normal. Neuroimaging and inflammatory workup results were within normal limits. An additional +1.00 lens improved her symptoms and visual acuities. Collaborative management involving psychiatry, neurology, and ophthalmology, including psychotherapy, led to significant symptom improvement. At the five-month follow-up, the patient experienced a complete resolution of her visual symptoms. Although Streff syndrome is a primary visual problem, additional psychogenic factors may add to variable cases. This case underscores the importance of recognizing stress-induced psychogenic manifestation, particularly in patients with underlying mental health conditions, and emphasizes an interdisciplinary management approach.

## Introduction

Streff syndrome was first reported by an optometrist, Dr. John Streff, in 1962 as a non-malingering, primary visual problem. This syndrome delineates characteristics found in non-malingering individuals with diminished visual acuity (more pronounced at near), reduced stereopsis, significant accommodative lag observed through dynamic retinoscopy, and constricted visual field [1,2]. It primarily affects the pediatric population, affecting the accommodative system in response to near-work stressors, which can lead to psychological issues like visual field defects and color vision problems. Application of a low-plus lens, typically ranging from +0.25 to +0.75, often results in immediate improvement of vision, fixation, and behavior, although it does not affect visual field or color vision [3]. However, there is a notable association between perceived stress environment and resultant anxiety, often causing a non-organic visual disturbance without structural damage [4]. While traditionally observed in younger individuals, this case report presents an unusual occurrence in a middle-aged woman with major depressive disorder. Herein, we present a case report of Streff-like syndrome in an adult woman, highlighting the importance of recognizing psychogenic manifestation and interdisciplinary management strategies.

## Case presentation

A 47-year-old woman with underlying major depressive disorder (MDD) presented with a range of symptoms, including bilateral blurring of vision, headache, and neurological symptoms, for one week following a stressful work-related incident. The blurring of vision was noticeable at a near distance, with an additional perceived loss of peripheral vision, which affected her work. Otherwise, there was no accompanying eye redness, eye pain, or any limitation in eye movement. The headache persisted primarily in the posterior region, with a severity of seven out of 10, exacerbated by loud noises and a bright environment. The pain was unresponsive to painkillers and tended to reoccur upon recollection of the triggering event. Additionally, the headache was accompanied by mild weakness and numbness in the right arm and leg. Otherwise, there was neither nausea, vomiting, or neck pain, and no hallucinations nor suicidal thoughts. Socially, she was a working single mother.

**Figure 1:**
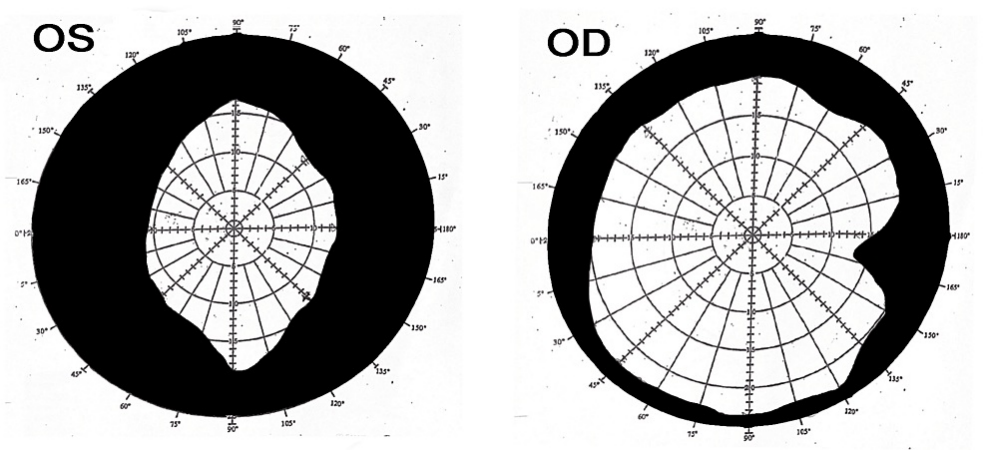
Bjerrum visual field test revealed constrictricted visual field both on the right eye (OD) and the left eye (OS).

**Figure 2:**
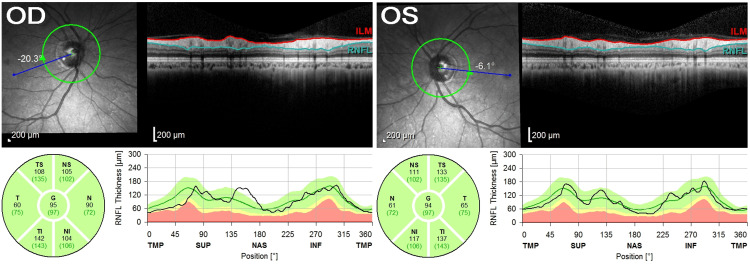
Optical coherence tomography (OCT) of the retinal nerve fibre layer (RNFL) thickness at the optic disc showed normal findings.

**Figure 3:**
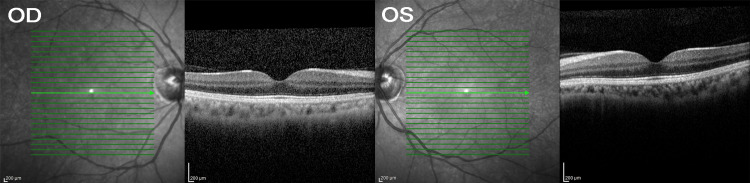
Optical coherence tomography (OCT) of macula was done due to near vision symptoms, no macula oedema observed.

On examination, her best corrected visual acuity was 6/21 OU. There was no relative afferent pupillary defect. Following the failure of the Ishihara test, a Hardy Rand and Ritter (HRR) test was done and showed an unclassified defect of medium tritan OD and red-green OS. Otherwise, light brightness was full and fluctuated during follow-ups. The anterior and posterior segment examinations revealed normal findings with no disc swelling or hyperemia. Bjerrum visual field test revealed a constricted visual field (Figure 1), which was tubular in pattern. Refraction showed improvement of vision with an additional +1.0 diopters (D) lens corrected to 6/6 and N5 bilaterally. Optical coherence tomography (OCT) of the optic disc retinal nerve fiber layer thickness and the macula revealed normal findings (Figures 2-3).

Extensive investigations were conducted to explore the patient's accompanying neurological symptoms. Contrasted computed tomography (CECT) of the brain and orbit exhibited normal results. Magnetic resonance imaging (MRI) of the brain and spine utilizing a demyelinating protocol revealed nonspecific T2-weighted flair hyperintensity at the right centrum semiovale without any associated active features. Other blood investigations, including a full blood count (FBC), erythrocyte sedimentation rate (ESR), rheumatoid factor (RF), C3, C4, antineutrophil cytoplasmic antibodies (ANCAs), and thyroid function test were within normal parameters.

The patient underwent collaborative interdisciplinary management involving teams from neurology, psychiatry, and ophthalmology, complemented by psychotherapy, resulting in significant improvement of her symptoms. Treatment included initiating oral Naproxen 550 mg PRN alongside the continuation of existing psychiatry medications: oral escitalopram 20 mg ON, oral olanzapine 15 mg ON, and oral lorazepam 1 mg PRN. Through ongoing monitoring with the ophthalmology team, there were fluctuations in visual acuity, color vision, light brightness, red saturation, and visual field. These aspects gradually improved, ultimately leading to a complete resolution of her visual symptoms by the five-month follow-up. Finally, the patient achieved a vision of 6/6 without the need for a new glass prescription.

## Discussion

In their 1994 literature review, Erickson et al. delved into the distinctions between malingering, hysterical amblyopia, and Streff syndrome [1]. The literature discussed how diagnosing these conditions involves distinguishing their underlying causes, particularly between Streff syndrome, mainly characterized by an autonomic nervous system disorder triggered by close work, and hysterical amblyopia, which originates from psychological factors. In clinical practice, Streff syndrome may exhibit additional psychogenic origin, as evidenced in this case, resulting in overlapping characteristics among patients.

In evaluating the visual fields of individuals diagnosed with Streff syndrome, clinicians can employ frequency-doubling technology, which helps uncover a uniform and extensive decrease in peripheral fields, frequently inherent in tubular patterns. This occurrence is thought to result from dysfunction in the magnocellular (M) pathway. Additionally, the relief of symptoms through the application of low-plus therapeutic lenses may be linked to the activation of ambient vision via the M pathway [5].

Generally, the American Academy of Ophthalmology (AAO), along with other organizations such as the American Academy of Pediatric Ophthalmology, the American Academy of Optometry, or the American Academy of Pediatrics, does not officially recognize the term Streff syndrome. Instead, the AAO utilized the term non-organic visual loss (NOVL) when discussing similar phenomena such as *hysterical*, *functional*, or *psychogenic* visual loss. In this case, the visual disturbances appear to stem from psychogenic causes impacting various aspects of vision, including the visual field, light brightness, red saturation, color vision, and near vision, which eventually improved with a plus lens +1.0 D. Although the diagnosis of Streff syndrome may be controversial, it is widely acknowledged and supported within the field of behavioral optometry [6].

In this case study, our findings indicated the importance of utilizing the term NOVL secondary to psychogenic cause to mitigate potential confusion. The term Streff syndrome was initially introduced to describe the difficulties in near work, which could lead to reduced visual field and color vision issues. Treatment modalities for Streff syndrome commonly involve the use of low-plus lenses. Given the variability and overlapping characteristics observed in these clinical presentations, employing a standardized terminology is crucial for accurate diagnosis and management. Furthermore, our findings emphasize the importance of comprehensive social history assessment, which may have lifesaving implications.

While mood disturbances are central to MDD, the disorder frequently manifests with various physical symptoms, such as headaches, as seen in our patient. The onset of visual disturbances and headaches following a stressful event suggests a psychogenic etiology, emphasizing an interconnected relationship between mental health and physical well-being. The accompanying neurological deficits further highlight the complex symptomatology observed in MDD. Hence, in this case, meticulous examination and investigations were done to rule out organic causes of visual impairment and headaches.

Psychogenic visual disturbance has been frequently linked to environmental stressors such as family and workplace conflicts, psychological distress, and preexisting physical illnesses. However, the prognosis is favorable upon the elimination of these stressors [7,8]. In this case study, the patient was able to overcome the symptoms largely due to the acknowledgment of her condition, which included a lack of social support and struggles with peer socialization. Her active participation during psychotherapy sessions and regular psychiatric clinic follow-ups greatly facilitated her emotional adjustment.

The treatment approach for MDD involves a combination of pharmacological and psychotherapeutic interventions to address its multifaceted nature and associated physical symptoms. In pediatric cases of Streff syndrome, management options may include low-plus eyewear such as reading glasses or multifocal, syntonic light therapy, and vision therapy aimed at enhancing peripheral awareness, focusing, and eye movement skills, which could also be applied to adult cases [2,5]. Additionally, offering reassurance and establishing a strong rapport with adult patients is beneficial, potentially enhancing their response to interventions [9,10]. Physicians can support patients by explaining that despite significant visual disturbances, investigations have not revealed any serious underlying ocular disease. Moreover, a study suggests that females presented with somatic symptoms, as observed in this case, may warrant additional attention for intervention [11].

## Conclusions

This case highlights the potential for external psychogenic factors to mimic Streff syndrome. Additionally, it emphasizes the importance of identifying stress-induced psychogenic manifestations in patients diagnosed with MDD, underscoring the necessity for interdisciplinary management strategies. Moreover, such manifestations are typically resolved spontaneously and associated with a positive visual prognosis.
